# Prognostic value of platelet-to-lymphocyte ratios among critically ill patients with acute kidney injury

**DOI:** 10.1186/s13054-017-1821-z

**Published:** 2017-09-08

**Authors:** Chen-Fei Zheng, Wen-Yue Liu, Fang-Fang Zeng, Ming-Hua Zheng, Hong-Ying Shi, Ying Zhou, Jing-Ye Pan

**Affiliations:** 1 Department of Nephrology, the First Affiliated Hospital of Wenzhou Medical University, Wenzhou, 325000 China; 2 School of the First Clinical Medical Sciences, Wenzhou Medical University, Wenzhou, 325000 China; 3 Department of Epidemiology, School of Basic Medical Sciences, Jinan University, Guangzhou, 510632 China; 4 Guangdong Provincial Key Laboratory of Food, Nutrition, and Health, School of Public Health, Sun Yat-sen University, Guangzhou, 510000 China; 5 Department of Infection and Liver Diseases, Liver Research Center, The First Affiliated Hospital of Wenzhou Medical University, Wenzhou, 325000 China; 6 Department of Preventive Medicine, Wenzhou Medical University, Wenzhou, 325000 China; 7 Department of Nephrology, The First Affiliated Hospital, Sun Yat-sen University, Key Laboratory of Nephrology, Ministry of Health and Guangdong Province, Guangzhou, 510000 China; 8 Department of Intensive Care, The First Affiliated Hospital of Wenzhou Medical University, Wenzhou, 325000 China

**Keywords:** Platelet-to-lymphocyte ratio, Acute kidney injury, Prognosis, Intensive care unit

## Abstract

**Background:**

Inflammation plays an important role in the initiation and progression of acute kidney injury (AKI). However, evidence regarding the prognostic effect of the platelet-to-lymphocyte ratio (PLR), a novel systemic inflammation marker, among patients with AKI is scarce. In this study, we investigated the value of the PLR in predicting the outcomes of critically ill patients with AKI.

**Methods:**

Patient data were extracted from the Multiparameter Intelligent Monitoring in Intensive Care Database III version 1.3. PLR cutoff values were determined using smooth curve fitting or quintiles and were used to categorize the subjects into groups. The clinical outcomes were 30-day and 90-day mortality in the intensive care unit (ICU). Cox proportional hazards models were used to evaluate the association between the PLR and survival.

**Results:**

A total of 10,859 ICU patients with AKI were enrolled. A total of 2277 thirty-day and 3112 ninety-day deaths occurred. A U-shaped relationship was observed between the PLR and both 90-day and 30-day mortality, with the lowest risk being at values ranging from 90 to 311. The adjusted HR (95% CI) values for 90-day mortality given risk values < 90 and > 311 were 1.25 (1.12–1.39) and 1.19 (1.08–1.31), respectively. Similar trends were observed for 30-day mortality or when quintiles were used to group patients according to the PLR. Statistically significant interactions were found between the PLR and both age and heart rate. Younger patients (aged < 65 years) and those with more rapid heart rates (≥89.4 beats per minute) tended to have poorer prognoses only when the PLR was < 90, whereas older patients (aged ≥ 65 years) and those with slower heart rates (<89.4 beats per minute) had higher risk only when the PLR was > 311 (*P* < 0.001 for age and *P* < 0.001 for heart rate).

**Conclusions:**

The preoperative PLR was associated in a U-shaped pattern with survival among patients with AKI. The PLR appears to be a novel, independent prognostic marker of outcomes in critically ill patients with AKI.

## Background

More than 5 million patients are admitted to intensive care units (ICUs) each year in America [1, 2], and 6–24% of these patients have acute kidney injury (AKI) [3]. In the presence of AKI, patient mortality increases to as high as 60–70%, especially within 1 year after ICU admission [4–6]. Considering the high incidence of AKI in the ICU and its poor prognosis, an increasing number of observational studies over the past 2 decades have been devoted to identifying the clinical predictors of mortality in AKI.

Systemic inflammation is an integral part of disease progression in critical illness and is commonly associated with sepsis, leading to an increased risk of mortality [7, 8]. Inflammation plays an important role in the initiation and progression of AKI [9–11], and morphological and/or functional changes in vascular endothelial cells and/or in the tubular epithelium are observed in patients with AKI. Leukocytes, including lymphocytes, infiltrate the injured kidneys and the entire body via the circulatory system and induce the generation of inflammatory mediators such as cytokines and chemokines, which damage the kidney and other organs [12]. The antithrombotic effects of platelets can evolve into atherogenesis via the secretion of proinflammatory cytokines [13], whereas the binding of platelets to endothelial cells can trigger leukocyte transmigration and adhesion, especially in the presence of shear stress [14]. The platelet-to-lymphocyte ratio (PLR) has been introduced as a potential marker of inflammation in cardiovascular disease (CVD) and tumors, which are also inflammation-related diseases [15–18]. A positive monotonic association between a high PLR and a poor prognosis for these diseases has been reported [15–18]. On the basis of the results of these studies, it is reasonable to speculate that the PLR might affect the prognosis of AKI. However, to the best of our knowledge, no epidemiological study to date has explored the prognostic effect of the PLR in patients with AKI.

## Methods

### Data source

This study was based on the publicly and freely available database known as the Multiparameter Intelligent Monitoring in Intensive Care Database III version 1.3 (MIMIC-III v1.3). This database comprises de-identified health-related data associated with over 40,000 patients treated in a variety of critical care units at Beth Israel Deaconess Medical Center (Boston, MA, USA) between 2001 and 2012 [15]. To apply for permission to access the database, researchers must complete the National Institutes of Health’s web-based course known as Protecting Human Research Participants (certification number 1605699).

The establishment of this database was approved by the institutional review boards of Massachusetts Institute of Technology (MIT, Cambridge, MA, USA) and Beth Israel Deaconess Medical Center. All included patients were de-identified to protect their privacy.

### Population selection criteria

A total of 58,976 ICU admissions were recorded in the MIMIC-III database. Eligible patients were those who were older than 18 years of age at first admission and who stayed in the hospital > 2 days. Patients were excluded from our study if (1) > 5% of their individual data were missing and (2) outliers were present. Outliers were defined as values exceeding the mean ± 3 times the SD.

The occurrence of AKI was determined on the basis of Kidney Disease: Improving Global Outcomes (KDIGO) classification [19], which specifies that serum creatinine (SCr) changes ≥ 1.5 times baseline must have occurred within the prior 7 days; a 0.3 mg/dl increase in SCr must have occurred within a 48-h period; and urine output must be < 0.5 ml/kg/h per 6 h. Stage 1 is defined as an increase in SCr to a level ≥ 1.5 times baseline or 0.3 mg/dl or urine output < 0.5 ml/kg/h per 6 h. Stage 2 is defined as an increase in SCr to a level ≥ 2.0 times baseline or urine output < 0.5 ml/kg/h per 12 h. Stage 3 is defined as an increase in SCr to a level ≥ 3.0 times baseline, an increase in SCr to a level ≥ 4.0 mg/dl, the initiation of renal replacement therapy (RRT), or urine output < 0.5 ml/kg/h per 12 h. Urine output was observed for the first 24 h after ICU admission. For patients who did not have an available SCr value prior to hospitalization, we followed the recommendation of the International Club of Ascites and used the first measured value during hospitalization as the baseline SCr [20].

### Date extraction

Patient data were exacted from MIMIC-III using Structured Query Language (SQL) with MySQL tools (version 5.6.24). The extracted data, including patient identifiers, demographic parameters, clinical parameters, laboratory parameters, and scoring systems, were collected from 2001 to 2012 at Beth Israel Deaconess Medical Center. Records containing baseline characteristics were extracted within the first 24 h after patient admission.

Laboratory measurements included platelets, white blood cells, lymphocytes, neutrophils, SCr levels, blood urea nitrogen (BUN) levels, serum potassium levels, serum sodium levels, serum pH, partial pressure of carbon dioxide, partial pressure of oxygen (PO_2_), serum glucose levels, and urine output. The PLR was defined as the ratio of the absolute platelet count to the absolute lymphocyte count.

Severity-of-illness scores, including the Simplified Acute Physiology Score II (SAPS II), Sequential Organ Failure Assessment (SOFA) score, and Glasgow Coma Scale (GCS) score, were recorded and calculated for each patient. In addition, the Elixhauser comorbidity score was used as a comorbidity estimate. Three other standard scoring systems were evaluated, enabling a comparison with our SAPS II score with glucose variability parameters (SOFA score, and Elixhauser comorbidity score). All scores were calculated using physiological measurements and clinical information according to published recommendations and accepted formulae.

The start date for follow-up was the date of the patient’s admission. The date of death was obtained from Social Security Death Index records from the U.S. government. All patients were followed for at least 3 months. The outcomes of our study were defined as 30-day and 90-day mortality.

### Statistical analysis and modeling strategy

Baseline characteristics were grouped by PLR cutoffs and are presented as frequency (percent) for categorical data and as mean (SD) or IQR for continuous data. Comparisons between groups were made using the chi-square test for categorical variables and analysis of variance or the Kruskal-Wallis test for continuous variables. Survival curves were generated using the Kaplan-Meier method and compared using the log-rank test. Cox proportional hazards models were used to test the associations between 90-day mortality (primary outcome) and baseline covariates, with results presented as HRs with 95% CIs. We also analyzed associations between the PLR and 30-day mortality. To determine whether the PLR was independently associated with endpoints, we performed multivariable analysis using a forward selection modeling process.

For each endpoint, two multivariate models were constructed on the basis of PLR group inclusion according to quintiles or cutoffs derived with curve-fitting methods based on 90-day mortality. The second quartile or the lower-limit group was treated as the reference group. In model 1, covariates were adjusted only for age and sex; in model 2, we further adjusted for PO_2_, ethnicity, GCS score, vasopressin use, ventilator use, systolic blood pressure (SBP), cardiac arrhythmias, valvular disease, pulmonary circulation, chronic pulmonary disease, liver disease, lymphoma, solid tumors, deficiency anemia, heart rate, SBP, potassium, SCr, urine output, BUN, and ph. We conducted stratification analyses to examine whether the effect of the PLR differed across various subgroups classified by AKI stage, RRT use, age, sex, ethnicity, PO_2_, GCS, heart rate, SBP, potassium, SCr, urine output, BUN, vasopressin use, ventilator use, comorbidities (i.e., cardiac arrhythmias, valvular disease, pulmonary circulation, chronic pulmonary disease, liver disease, lymphoma, metastatic cancer, solid tumors, and deficiency anemias), and cardiac surgery. Multiplicative interactions were estimated by adding interaction terms according to the likelihood ratio test. All statistical analyses were performed using the IBM SPSS Statistics version 19.0 (IBM, Armonk, NY, USA), EmpowerStats (http://www.empowerstats.cn/), and MedCalc (MedCalc Software, Ostend, Belgium) software programs. A two-tailed *P* value < 0.05 was considered statistically significant.

## Results

### Subject characteristics

Patient records from 14,354 subjects who underwent ICU treatment at Beth Israel Deaconess Medical Center were initially extracted from the MIMIC-III database. After patients who did not meet the inclusion criteria were excluded, 10,859 eligible subjects were enrolled. The subjects included 5931 men and 4928 women with a mean age of 65.4 (15.8) years. Of these subjects, 6881 (63.4%) patients were recruited from the medical ICU, and 3978 (36.6%) patients were recruited from the surgical ICU.

**Fig. 1:**
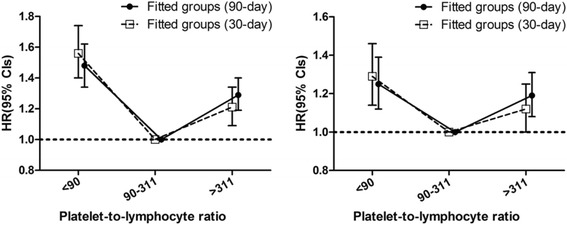
HRs (95% CIs) for all-cause mortality across fitted groups of platelet-to-lymphocyte ratios (fitted groups: model 1 and model 2)

The overall mean (SD) PLR was 285.7 (256.9). When the patients were divided on the basis of 90-day mortality according to the curve-fitting method (Fig. 1), 1708 (15.7%) were in the low-PLR group (PLR < 90), 6699 (22.6%) were in the mid-PLR group (90–311), and 2454 (22.6%) were in the high-PLR group (PLR > 311). Selected characteristic and hematologic laboratory data across PLR groupings are provided in Table 1. Participants with higher calibrated PLRs (PLR > 311) were more likely to be elderly, female, and white and to report a history of chronic pulmonary disease, metastatic cancer, solid tumors, and iron-deficiency anemia; they also had higher levels of serum potassium, BUN, white blood cells, neutrophils, platelets, urine output, and eGFR and were more likely to use RRT than those with lower PLRs (PLR < 90).

**Table 1:** Baseline characteristics of the study population

Characteristics	Platelet-to-lymphocyte ratio			*P* value
	<90	90–311	≥312	
Platelet-to-lymphocyte ratio	59.1	182.6	456.8	<0.001
Clinical parameters, *n* (%)	1708	6699	2454	
Age, years	62.3 (15.9)	65.4 (15.9)	65.7 (15.5)	<0.001
Sex, *n* (%)				<0.001
Male	992 (58.1)	3679 (54.9)	1260 (51.3)	
Female	716 (41.9)	3018 (45.1)	1194 (48.7)	
Ethnicity, *n* (%)				<0.001
White	1095 (64.10)	4636 (69.2)	1833 (74.7)	
Black	203 (11.9)	805 (12.0)	208 (8.5)	
Other	410 (24.0)	1256 (18.8)	413 (16.8)	
SBP, mmHg	120.6 (26.4)	123.3 (26.3)	121.9 (26.0)	<0.001
DBP, mmHg	61.5 (18.6)	62.2 (18.1)	61.1 (17.3)	0.021
Heart rate, beats/minute	89.9 (20.9)	88.6 (19.9)	91.3 (20.0)	<0.001
Vasopressin use, *n* (%)				<0.001
Yes	844 (49.4)	2728 (40.7)	1047 (42.7)	
No	864 (50.6)	3969 (59.3)	1407 (57.3)	
Ventilator use, *n* (%)				<0.001
Yes	1063 (62.2)	3528 (52.7)	1316 (53.6)	
No	645 (37.8)	3169 (47.3)	1138 (6.4)	
Comorbidities				
Cardiac arrhythmias, *n* (%)				<0.001
Yes	375 (22.0)	1944 (29.0)	696 (28.4)	
No	1333 (78.0)	4753 (71.0)	1758 (71.6)	
Valvular disease, *n* (%)				0.051
Yes	177 (15.7)	740 (11.0)	228 (9.3)	
No	1531 (89.6)	5957 (89.0)	2226 (90.7)	
Pulmonary circulation, *n* (%)				0.599
Yes	97 (5.7)	415 (6.2)	158 (6.4)	
No	1611 (94.3)	6282 (93.8)	2296 (93.6)	
Chronic pulmonary disease, *n* (%)				<0.001
Yes	255 (14.9)	1343 (20.1)	558 (22.7)	
No	1453 (85.1)	5354 (79.9)	1896 (77.3)	
Liver disease, *n* (%)				<0.001
Yes	300 (17.6)	492 (7.3)	108 (4.4)	
No	1408 (82.4)	6205 (92.7)	2346 (95.6)	
Lymphoma, *n* (%)				<0.001
Yes	59 (3.5)	123 (1.8)	49 (2.0)	
No	1649 (96.5)	6574 (98.2)	2405 (98.0)	
Metastatic cancer, *n* (%)				<0.001
Yes	62 (3.6)	280 (4.2)	203 (8.3)	
No	1646 (96.4)	6417 (95.8)	2251 (91.7)	
Solid tumor, *n* (%)				<0.001
Yes	93 (5.4)	411 (6.1)	248 (10.1)	
No	1615 (94.6)	6286 (93.9)	2206 (89.9)	
Deficiency anemias, *n* (%)				0.046
Yes	246 (14.4)	1014 (15.1)	416 (17.0)	
No	1462 (85.6)	5683 (84.9)	2038 (83.0)	
Laboratory parameters				
PO_2_, mmHg	118.0 (74.0–279.0)	113.0 (75.0–237.0)	107.0 (72.3–201.0)	<0.001
PCO_2_, mmHg	39.0 (34.0–46.0)	40.0 (34.0–47.0)	40.0 (34.0–47.0)	0.036
Serum potassium, mmol/L	4.32 (0.89)	4.39 (0.91)	4.44 (0.95)	<0.001
BUN, mg/dl	30.0 (20.0–47.0)	31.0 (21.0–49.0)	33.0 (21.0–52.0)	<0.001
Serum bicarbonate, mmol/L	23.0 (19.0–26.0)	24.0 (20.0–27.0)	23.0 (20.0–27.0)	<0.001
Serum pH	7.31 (0.14)	7.32 (0.12)	7.32 (0.12)	<0.001
White blood cell count, 10^9^/L	11.0 (7.4–16.5)	10.6 (7.6–15.1)	11.8 (8.1–16.5)	<0.001
Neutrophil count, *n* (%)	65.9 (18.2)	77.6 (11.4)	83.5 (12.9)	<0.001
Lymphocyte count, *n* (%)	19.0 (12.0–28.0)	12.0 (8.0–17.7)	5.9 (3.8– 8.8)	<0.001
Platelet count, 10^9^/L	122.0 (67.0–182.)	220.0 (164.0–287.0)	285.0 (206.0–383.8)	<0.001
Scoring systems				
GCS	14.0 (6.0–15.0)	15.0 (8.0–15.0)	15.0(9.0–15.0)	<0.001
SOFA	7.0 (5.0–10.0)	5.0 (3.0– 8.0)	5.0 (3.0– 8.0)	<0.001
Renal function				
Serum creatinine, mg/dl	1.50 (1.10–2.40)	1.60 (1.20–2.40)	1.60 (1.20–2.50)	0.340
Urine output, ml/24 h	1050 (372–2311)	1050 (395–2325)	900 (345–2095)	0.046
eGFR, ml/min/1.73 m^2^	0.88 (0.76–0.94)	0.87 (0.77–0.95)	0.84 (0.76–0.94)	<0.001
eGFR change	2.02 (1.17–4.27)	1.15 (1.86–3.76)	2.03 (1.25–4.16)	<0.001
KDIGO stage, *n* (%)				0.084
Stage 1	1081 (63.4)	4467(66.7)	1589 (64.8)	
Stage 2	407 (23.9)	1456 (21.8)	573 (23.3)	
Stage 3	218 (12.8)	776 (11.6)	292 (11.9)	
Renal replacement therapy				0.008
Yes	71 (4.2)	189 (2.8)	66 (2.7)	
No	1635 (95.8)	6510 (97.2)	2388 (97.3)	

*Abbreviations: BUN* Blood urea nitrogen, *DBP* Diastolic blood pressure, eGFR, Estimated glomerular filtration rate; *GCS* Glasgow Coma Scale, *KDIGO* Kidney Disease: Improving Global Outcomes, *PCO*_*2*_ Partial pressure of carbon dioxide, *PO*_*2*_ Partial pressure of oxygen, *SBP* Systolic blood pressure, *SOFA* Sequential Organ Failure Assessment

Normally distributed data are presented as the mean (SD) (analysis of variance); non-normally distributed data are presented as median (IQR) (nonparametric Wilcoxon test); and categorical variables are presented as *n* (%) (chi-square test)

### Association between platelet-to-lymphocyte ratio and 30-day and 90-day outcomes

**Fig. 2:**
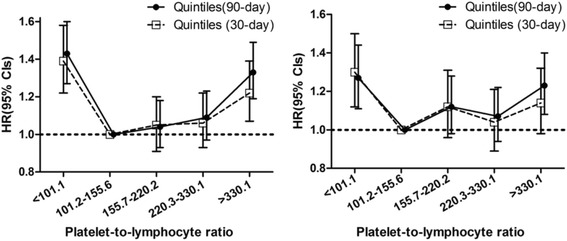
HRs (95% CIs) for all-cause mortality across quintile groups of platelet-to-lymphocyte ratios (quintile groups: model 1 and model 2)

A total of 2277 thirty-day and 3112 ninety-day deaths occurred during the follow-up period. A U-shaped relationship was observed between the PLR and 90-day mortality, and the patients in the mid-PLR group (90–311) had the lowest 30-day mortality rate when compared with rates in the PLR < 90 and PLR > 311 groups (both *P* < 0.001). The adjusted HRs (95% CIs) for PLRs < 90 and > 311 were 1.25 (1.12–1.39) and 1.19 (1.08–1.31), respectively. A similar trend was observed for 30-day mortality, and the risk was less evident with higher PLRs (*P* = 0.047 for PLR > 311). Following the stratification of PLRs into quintiles and using the second quintile (PLR 101.2–155.6) as a reference, both extremely low (<101.2) and extremely high (>330.2) PLRs were associated with an increased risk of 90-day mortality. For 30-day mortality, the lowest PLRs (<101.2, *P* = 0.001) were associated with an increased risk, whereas marginally increased risk was associated with extremely high PLRs (>330.2, *P* = 0.088) after adjustment for potential confounders (Figs. 1 and 2 and Table 2).

**Table 2:** HRs (95% CIs) for all-cause mortality across groups of platelet-to-lymphocyte ratios

Platelet-to-lymphocyte ratio	No. of patients/deaths	Model 1		Model 2	
		HR (95% CIs)	*P* value	HR (95% CIs)	*P* value
90-Day all-cause mortality					
Fitted groups					
< 90	1708/573	1.48 (1.34–1.62)	<0.001	1.25 (1.12–1.39)	<0.001
90–311	6697/1745	1.00		1.00	
≥ 312	2454/794	1.29 (1.19–1.40)	<0.001	1.19 (1.08–1.31)	<0.001
Quintiles					
< 101.1	2172/694	1.43 (1.27–1.60)	<0.001	1.27 (1.11–1.44)	<0.001
101.2–155.6	2172/545	1.00		1.00	
155.7–220.2	2173/571	1.04 (0.93–1.18)	0.477	1.12 (0.98–1.28)	0.093
220.3–330.1	2171/602	1.09 (0.97–1.23)	0.152	1.07 (0.94–1.22)	0.296
≥ 330.2	2171/700	1.33 (1.19–1.49)	<0.001	1.23 (1.08–1.40)	0.002
30-Day all-cause mortality					
Fitted groups					
< 90	1708/448	1.56 (1.40–1.74)	<0.001	1.29 (1.14–1.46)	<0.001
90–311	6697/1273	1.00		1.00	
≥ 312	2454/556	1.21 (1.09–1.34)	<0.001	1.12 (1.00–1.25)	0.047
*P*_trend_					
Quintiles					
< 101.1	2172/532	1.39 (1.22–1.58)	<0.001	1.30 (1.12–1.50)	0.001
101.2–155.6	2172/406	1.00			
155.7–220.2	2173/423	1.05 (0.91–1.20)	0.516	1.12 (0.96–1.31)	0.141
220.3–330.1	2171/432	1.06 (0.93–1.22)	0.388	1.04 (0.89–1.21)	0.649
≥ 330.2	2171/484	1.22 (1.07–1.39)	0.003	1.14 (0.98–1.32)	0.088

Models 1 and 2 were derived from Cox proportional hazards regression models: model 1 covariates were adjusted for age and sex; model 2 covariates were adjusted for age, sex, partial pressure of oxygen, ethnicity, Glasgow Coma Scale score, vasopressin use, ventilator use, systolic blood pressure, cardiac arrhythmias, valvular disease, pulmonary circulation, chronic pulmonary disease, liver disease, lymphoma, solid tumor, deficiency anemias, heart rate, potassium creatinine, urine output, blood urea nitrogen, and pH

### Subgroup analyses

In the subgroup analyses, the association between the PLR and the risk of 90-day mortality was similar for most strata (*P* = 0.083–0.983) (Table 3). Significant interactions were observed only for age (*P* < 0.001). Patients younger than 65 years of age had a significantly higher risk of 90-day mortality for a PLR < 90 (HR 1.37, 95% CI 1.16–1.62, *P* < 0.001), whereas only older patients (≥65 years) showed an increased risk for a PLR > 311 (HR 1.25 95% CI 1.22–1.40, *P* < 0.001). Similarly, patients with a heart rate < 89.4 beats per minute had a significantly higher risk of 90-day mortality with a PLR > 311 (HR 1.29, 95% CI 1.22–1.48, *P* < 0.001), whereas patients with a heart rate ≥ 89.4 beats per minute (aged ≥ 65 years) showed an increased risk only with a PLR < 90 (HR 1.39, 95% CI 1.20–1.61, *P* < 0.001).

**Table 3:** Subgroup analysis of the associations between 90-day all-cause mortality and the platelet-to-lymphocyte ratio

	No. of patients/deaths	Platelet-to-lymphocyte ratio			
		<90	90–311	≥312	*P* for interaction
AKI stage					0.301
Stage 1	7137/1740	1.23 (1.05–1.43)^a^	1.00	1.22 (1.08–1.38)^a^	
Stage 2	2436/1474	1.23 (1.02–1.50)^b^	1.00	1.09 (0.92–1.30)	
Stage 3	1286/876	1.38 (1.04–1.82)	1.00	1.30 (0.99–1.72)	
Renal replacement therapy					0.906
Yes	326/177	1.45 (0.97–2.17)	1.00	1.31 (0.86–2.01)	
No	10,533/2935	1.24 (1.11–1.39)^c^	1.00	1.17 (1.06–1.29)^a^	
Age, years					<0.001
< 65	4686/1000	1.37 (1.16–1.62)^c^	1.00	1.06 (0.88–1.27)	
≥ 65	6035/2054	1.09 (0.94–1.27)	1.00	1.25 (1.12–1.40)^c^	
Sex					0.386
Male	5931/1741	1.22 (1.06–1.41)^a^	1.00	1.24 (1.09–1.41)^c^	
Female	4928/1371	1.26 (1.06–1.49)^a^	1.00	1.13 (0.98–1.31)	
Ethnicity					0.409
White	7564/2134	1.25 (1.09–1.44)^c^	1.00	1.19 (1.06–1.33)^a^	
Black	1216/254	1.24 (0.84–1.81)	1.00	1.33 (0.92–1.93)	
Other	2079/724	1.21 (0.98–1.49)	1.00	1.11 (0.90–1.36)	
PO_2_, mmHg					0.310
< 167.3	6433/2108	1.29 (1.14–1.47)^c^	1.00	1.21 (1.08–1.35)^a^	
≥1 67.3	3502/870	1.16 (0.95–1.42)	1.00	1.21 (1.00–1.47)	
GCS score					0.938
< 11.4	4101/1387	1.25 (1.07–1.46)^a^	1.00	1.21 (1.05–1.39)	
≥ 11.4	6499/1663	1.24 (1.06–1.45)^a^	1.00	1.18 (1.04–1.35)^b^	
Heart rate, beats/min					0.001
< 89.4	5811/1503	1.08 (0.92–1.28)	1.00	1.29 (1.12–1.48)^c^	
≥ 89.4	4880/1575	1.39 (1.20–1.61)^c^	1.00	1.10 (0.96–1.26)	
SBP, mmHg					0.187
< 122.6	5596/1885	1.28 (1.12–1.46)^c^	1.00	1.15 (1.02–1.30)^b^	
≥ 122.6	4800/1070	1.18 (0.97–1.43)	1.00	1.26 (1.07–1.47)^a^	
Potassium, mmol/L					0.633
< 4.39	6158/1667	1.24 (1.07–1.44)^a^	1.00	1.19 (1.04–1.36)^a^	
≥ 4.39	4692/1443	1.24 (1.05–1.47)^b^	1.00	1.22 (1.06–1.40)^a^	
Creatinine, mg/dl					0.816
< 2.28	7809/2128	1.29 (1.13–1.47)^c^	1.00	1.21 (1.08–1.35)^a^	
≥ 2.28	3048/982	1.17 (0.96–1.44)	1.00	1.15 (0.97–1.37)	
Urine output, ml/24 h					0.083
< 1807	7057/2309	1.28 (1.13–1.45)^c^	1.00	1.13 (1.02–1.26)^b^	
≥ 1807	3300/641	1.17 (0.92–1.47)	1.00	1.38 (1.12–1.69)^a^	
BUN, mg/dl					0.615
< 39.2	6887/1737	1.30 (1.13–1.50)^c^	1.00	1.23 (1.08–1.40)^a^	
≥ 39.2	3972/1375	1.19 (1.00–1.41)	1.00	1.17 (1.01–1.35)^b^	
Vasopressin use					0.549
Yes	4619/1784	1.29 (1.13–1.48)^c^	1.00	1.24 (1.09–1.40)^a^	
No	6240/1328	1.17 (0.96–1.42)	1.00	1.11 (0.95–1.29)	
Ventilator use					0.216
Yes	5907/2098	1.26 (1.12–1.43)^c^	1.00	1.17 (1.05–1.31)^a^	
No	4952/1014	1.21 (0.95–1.54)	1.00	1.21 (1.01–1.45)^b^	
Cardiac arrhythmias					0.671
Yes	3015/1040	1.13 (0.91–1.39)	1.00	1.19 (1.02–1.40)^b^	
No	7844/2072	1.29 (1.13–1.47)^c^	1.00	1.19 (1.06–1.34)^a^	
Valvular disease					0.986
Yes	1145/339	0.75 (0.51–1.10)	1.00	1.37 (1.01–1.87)^b^	
No	9714/2773	1.31 (1.17–1.47)^c^	1.00	1.18 (1.07–1.31)^a^	
Pulmonary circulation					0.320
Yes	670/199	1.20 (0.76–1.89)	1.00	1.12 (0.76–1.63)	
No	10,189/2913	1.34 (1.11–1.39)^c^	1.00	1.18 (1.07–1.31)^a^	
Chronic pulmonary disease					0.289
Yes	2156/638	0.97 (0.72–1.29)	1.00	1.14 (0.94–1.38)	
No	8703/2474	1.29 (1.15–1.46)^c^	1.00	1.21 (1.08–1.34)^a^	
Liver disease					0.919
Yes	900/410	1.13 (0.89–1.45)	1.00	1.08 (0.78–1.50)	
No	9959/2702	1.26 (1.12–1.42)^c^	1.00	1.21 (1.09–1.33)^c^	
Lymphoma					0.101
Yes	231/102	1.66 (0.94–2.93)	1.00	1.79 (0.97–3.30)	
No	10,628/3010	1.23 (1.10–1.37)^c^	1.00	1.18 (1.07–1.30)^a^	
Metastatic cancer					0.901
Yes	545/317	0.97 (0.63–1.49)	1.00	1.11 (0.84–1.45)	
No	10,314/2795	1.26 (1.12–1.41)^c^	1.00	1.15 (1.04–1.27)^a^	
Solid tumor					0.605
Yes	752/333	1.22 (0.82–1.81)	1.00	1.21 (0.91–1.60)	
No	10,107/2779	1.24 (1.10–1.38)^c^	1.00	1.18 (1.07–1.31)^a^	
Deficiency anemias					0.852
Yes	1676/378	1.15 (0.91–1.46)	1.00	1.12 (0.77–1.53)	
No	9183/2734	1.25 (1.10–1.39)	1.00	1.20 (1.07–1.33)	
Cardiac surgery					0.207
Yes	1864/635	1.16 (0.92–1.45)	1.00	1.24 (1.01–1.52)^b^	
No	8995/2477	1.30 (1.14–1.47)^c^	1.00	1.16 (1.04–1.29)^a^	

*Abbreviations: AKI* Acute kidney injury, *BUN* Blood urea nitrogen, *GCS* Glasgow Coma Scale, *PO*_*2*_ Partial pressure of oxygen, *SBP* Systolic blood pressure

^a^ *P* < 0.01

^b^ *P* < 0.05

^c^ *P* < 0.001

HRs (95% CIs) were derived from Cox proportional hazards regression models. Covariates were adjusted as in model 2 (Table 2)

## Discussion

In this study, we observed a U-shaped relationship between the PLR and 30-day and 90-day mortality, and both low and high PLRs were associated with increased all-cause mortality. Proctor et al. [21] investigated the correlation between the PLR and overall survival in a large-scale cohort of 8759 patients with cancer. In contrast to our results, their study showed a positive correlation between the PLR and mortality when using a similar PLR cutoff (PLR < 150, HR 1; PLR 150–300, HR 1.19; *P* < 0.001; PLR > 300, HR 1.71, *P* < 0.001). Yaprak et al. [22] recently evaluated the correlation between the PLR and mortality in a small cohort of patients with end-stage renal disease (ESRD) and demonstrated that the PLR could independently predict all-cause mortality in this population. A main reason for this difference is the insufficient number of patients with low PLRs. However, our study addresses this limitation. In the Framingham Heart Study, blood cell composition was treated as a prognostic factor for CVD, and the association between hematocrit and CVD mortality showed a U-shaped association in both men and women [23].

Both AKI and chronic kidney disease (CKD) are associated with local and systemic inflammation [24]. Researchers in many observational studies have described high circulating levels of inflammatory mediators and adverse outcomes for these conditions. These inflammatory mediators include blood cells, components of endothelial cells, platelets, lymphocytes, macrophages, mast cells, and fibroblasts. The PLR has been investigated as a new inflammatory marker for predicting major adverse events associated with CVD [16]. In a study of 2563 patients, Velibey et al. [25] demonstrated that increased PLRs are independently associated with a greater risk of contrast-induced AKI in patients undergoing primary percutaneous coronary intervention. A recent study showed that a high PLR is related to the presence of coronary artery disease and is correlated with C-reactive protein and fibrinogen levels [26]. High PLRs in patients with ESRD were also associated with high levels of inflammation. Balta et al. [27] showed that inflammation is better predicted by the PLR than by the neutrophil-to-lymphocyte ratio in ESRD. On the basis of the association between PLR-related inflammation and disease severity, we speculated that excessively high PLRs could predict the same poor outcomes as other inflammation biomarkers in AKI populations.

In a population-based cohort study comparing the mortality rates of 605 ICU patients with those of patients with acute renal failure (ARF), Mehta et al. [28] found that a platelet count < 20,000/mm^3^ is a criterion for hematologic failure and that the risk of mortality was more than threefold higher among patients who had hematologic failure than among control patients with normal platelet levels (OR 3.39, 95% CI 2.08–5.52). Using data from the Program to Improve Care in Acute Renal Disease, a multicenter observational study of ARF, Chertow et al. [29] examined the correlates of mortality in 618 ICU patients with ARF. Thrombocytopenia was associated with mortality at the time of consultation. In addition, among 512 ICU patients requiring acute dialysis, a platelet count < 50,000/mm^3^ was a potential risk factor for mortality in multivariate analysis [30]. Thrombocytopenia is common among critically ill patients and is often associated with poor outcomes [31–34]. The mechanisms underlying thrombocytopenia include either reduced platelet production or excessive platelet destruction related to an underlying illness and therapeutic interventions [35]. Taken together, these findings show that low platelet counts could result from a low PLR in patients with AKI and could lead to high mortality, thus helping to explain our observation of a U-shaped relationship between the PLR and mortality.

Although AKI in the ICU is associated with high mortality, other factors appear to contribute to poor outcomes. Potential factors that may affect the outcome of AKI include blood pressure [36], renal function [37], urine output [36], and other clinical parameters (i.e., SCr, BUN, and pH [38]), as well as comorbidities (e.g., cardiac disease [38]). In the present study, when patients were stratified according to potential confounders, no significant interactions were observed for sex, ethnicity, PO_2_, GCS, SBP, potassium, SCr, urine output, BUN, vasopressin use, or ventilator use. Although possible interactions between all-cause mortality and both age and heart rate were observed, there was no heterogeneity of clinical factors among those effects and the PLR. However, little is known about the mechanism underlying the interaction between age and the PLR. Recently, in a multicenter cross-sectional study of a healthy Indian population, Sairam et al. [39] found lower levels of platelets in elderly people. Kweon et al. [40] investigated median PLRs in a healthy Korean population and suggested that the PLR cutoff values for disease evaluation should be established separately according to age. Therefore, we should consider age when evaluating the relationship between the PLR and mortality among critically ill patients with AKI.

The limitations of this study should be acknowledged. First, it was a single-center retrospective analysis, and different conclusions could be reached when using patient records from other centers. Therefore, subject selection bias cannot be ignored, suggesting that a prospective multicenter study is needed. However, the strength of the present study is its representative and ethnically diverse population. Second, because of a lack of data on kidney function prior to 3 months before patient admission, we could not assess CKD status among patients with AKI or determine the role of CKD in the association between the PLR and mortality. Third, the PLR can be measured in patients only upon admission to the ICU. A single measure of the PLR does not fully reflect inflammation, which would be better assessed by simultaneously measuring other inflammatory mediators. Fourth, these preliminary data suggest that the PLR could be a risk adjustment tool with prognostic implications for AKI. Fifth, to establish the PLR as a prognostic marker, researchers must further validate its clinical significance. The cutoff value must be established in one cohort of patients and tested in another, and the number of patients in each group needs to be considered in statistical analyses. Finally, we did not assess the modification of sepsis and shock, which both might increase patient morbidity and predict higher mortality among patients with AKI [41], on association between PLR and outcomes, owing to lack of related data.

## Conclusions

We found a U-shaped relationship between the PLR and mortality in which both low and high PLRs were associated with increased overall mortality in critically ill patients with AKI. The PLR is therefore potentially useful in the clinical setting as a cost-effective and readily available biomarker. Our findings need to be confirmed by other studies, especially large prospective studies with longer follow-up.

## Abbreviations

AKI: Acute kidney injury
ARF: Acute renal failure
BUN: Blood urea nitrogen
CKD: Chronic kidney disease
CVD: Cardiovascular disease
DBP: Diastolic blood pressure
eGFR: Estimated glomerular filtration rate
ESRD: End-stage renal disease
GCS: Glasgow Coma Scale
ICU: Intensive care unit
KDIGO: Kidney Disease: Improving Global Outcomes
MIMIC-III: Multiparameter Intelligent Monitoring in Intensive Care Database III
PCO_2_: Partial pressure of carbon dioxide
PLR: Platelet-to-lymphocyte ratio
PO_2_: Partial pressure of oxygen
RRT: Renal replacement therapy
SAPS II: Simplified Acute Physiology Score II
SBP: Systolic blood pressure
SCr: Serum creatinine
SQL: Structured Query Language
SOFA: Sequential Organ Failure Assessment
